# A Low-Cost Micro-Volume Nephelometric System for Quantitative Immunoagglutination Assays

**DOI:** 10.3390/s19204359

**Published:** 2019-10-09

**Authors:** Qiqi Sun, Wei Zheng, Chao Lin, Dongxuan Shen

**Affiliations:** 1 Research Laboratory for Biomedical Optics and Molecular Imaging, Shenzhen Key Laboratory for Molecular Imaging, Shenzhen Institutes of Advanced Technology, Chinese Academy of Sciences, Shenzhen 518055, China; zhengwei@siat.ac.cn; 2 Edan Instruments, Inc., Shenzhen 518067, China; linchao@edan.com.cn (C.L.); dshen@edan.com.cn (D.S.)

**Keywords:** immunoassay, immunoagglutination assay, light scattering, nephelometry, C-reactive protein

## Abstract

Immunoassays have been widely used in scientific research and clinical diagnosis due to their versatile detection capability and high specificity. Immunoagglutination assays are kinds of immunoassay, which can simply and rapidly measure the concentration of analytes. In this work, we developed a low-cost micro-volume nephelometric system for quantitative immunoagglutination assays. We used off-the-shelf components to build the system, and the total cost of key components is only about 20 US dollars. The total detection volume in our system was as low as 3 µL, which could significantly reduce the reagent cost and required sample volume. We further evaluated the system performance via the immunoagglutination assay to measure the concentration of C-reactive protein, a plasma protein with levels rising in response to inflammation. The results demonstrated that our system could measure the concentration of analytes with relatively high sensitivity and precision within four minutes, and has high potential to be applied for clinical diagnostic tests.

## 1. Introduction

Immunoassays are biochemical methods that measure the presence or concentration of analytes through highly specific binding between antigens and antibodies. Analytes detected by immunoassays could be molecules of different size and types, such as hormones, drugs and proteins. Due to their versatile detection capability, high sensitivity and specificity, immunoassay methods have been widely used in many important areas, such as diagnosis of diseases, therapeutic drug monitoring, clinical pharmacokinetic studies, and so on [1]. Generally, immunoassays can be divided into homogeneous and heterogeneous ones. In heterogeneous immunoassays, the antigen-antibody complexes are bound to a solid substrate and the unbound immune reagents are washed out of the system. Enzyme-linked immunosorbent assays (ELISA) are representatives of heterogeneous immunoassays, and tests based on them have served as the gold standard for a plethora of analytes [2]. Although heterogeneous immunoassays are more versatile and more sensitive (e.g., chemiluminescence immunoassays), they may require a longer run time and more complex manipulations [3]. In homogeneous immunoassays, such as immunoagglutination assays, the assay procedures do not need the separation of the antigen-antibody complexes from unbound immune reagents, indicating that they may be more favorable for rapid and cost-effective applications.

Developing low-cost, rapid and portable medical systems is significant to improve the accessibility of medical care, especially in regions of limited resources, since medical tests are generally slow and expensive, and require trained personnel and high-end equipment. Therefore, many studies have focused on reducing the cost of biomedical and clinical systems, such as the development of inexpensive imaging systems [4,5,6] and fluorescence-based detection systems [7,8]. Among immunoassay-based diagnostic tests, products based on lateral flow assays, such as the test of human chorionic gonadotropin used in pregnancy diagnosis, have been massively commercialized because they are fast, low-cost and easy-to-use. However, their results are mostly qualitative or semi-quantitative [9], which limits their further applications when quantitative measurement is prerequisite. Immunoagglutination assays are fast, simple, and quantitative methods to detect analytes in a fluid test sample [10,11]. In these methods, antigens and antibodies are crosslinked and aggregated to form large immune complexes, and latex particles, with the predominant type being polystyrene, are usually used as carriers of antigens or antibodies to enhance method sensitivity [11,12]. The procedures of immunoagglutination assays are simpler than heterogeneous immunoassays because only the mixing of samples and reagents is involved. Furthermore, in clinical applications, immunoagglutination assays could reduce the assay time from 3 h (ELISA) to 10 min [13], and have comparable performance to other heterogeneous immunoassays, such as ELISA [13] and microparticle enzyme immunoassay [14]. Therefore, it is suitable and significant to develop a rapid and low-cost immunoagglutination assay system for biomedical and clinical applications.

In immunoagglutination assay, the aggregation of antibody and antigen can be detected via electrical or optical methods. In electrical methods, complex electrodes and/or sophisticated microfluidic chips are usually used [15,16,17], which might hinder their mass production in practice. Turbidimetry and nephelometry are commonly used optical detection methods in immunoagglutination assays. Turbidimetry measures intensity of light transmitted through the sample with a certain thickness, usually 5 mm or 10 mm (length of light path). Nephelometry measures the light scattered by the sample and is less dependent on sample thickness, indicating that we have the potential to design a micro-volume nephelometric system. Many studies have focused on developing a rapid and portable immunoagglutination system based on optical detection method [18,19,20], but the use of microfluidic chips as detection cuvettes and/or optical systems with expensive components, such as spectrometers and avalanche photodiodes, are unsuitable for widespread application. In this work, we developed an innovative nephelometric system for quantitative immunoagglutination assays. We used off-the-shelf components to build the system with single-use glass capillary tubes as detection cuvettes. The total cost of key components of the system was only about 20 US dollars. Furthermore, the detection volume in our system was as low as 3 µL, which could significantly reduce the required sample volume and cost of reagents. Finally, we demonstrated that the system could quantitatively measure the concentration of C-reactive protein (CRP) with relatively high sensitivity and precision within four minutes. This indicated that the proposed system was capable of delivering fast and precise results for clinical diagnostic tests.

## 2. Materials and Methods

### 2.1. Nephelometric System Setup

**Figure 1:**
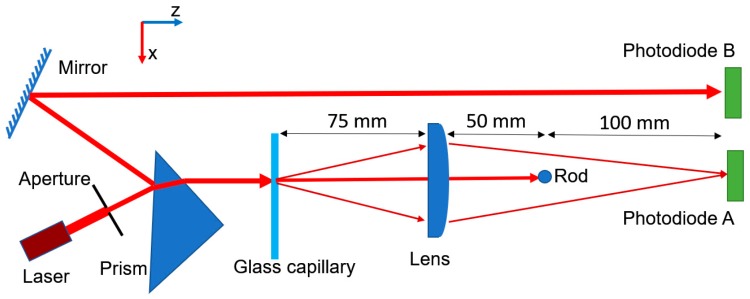
The schematic of nephelometric system. Red arrows indicate light path.

The schematic of nephelometric system is shown in Figure 1. A red laser module (Ruixinfeng Electronics Technology, Shenzhen, China) was employed as a light source. It consisted of a 670 nm wavelength laser diode, a constant current power supply board and collimation lenses. The collimated laser beam passed through a circular aperture with 3 mm diameter to decrease the size of laser spot, and the laser power measured after the aperture was ~0.5 mW. The light had an elliptical beam profile. The length of long axis was 3 mm, which was determined by the diameter of circular aperture, and the length of short axis was about 1.8 mm. The long axis of light was set parallel to the x-z plane of system. Then, a prism picked off ~10% of laser power via Fresnel reflection to monitor intensity fluctuation. The real-time fluctuation of laser power was recorded through photodiode B, as shown in Figure 1, and the nephelometric signal recorded by photodiode A was normalized to this laser fluctuation signal, so that the influence of laser instability could be minimized.

Next, the beam went through a glass capillary containing the mixture of sample and reagents to be analyzed. The light scattered by aggregations in the mixture was collected by a plano-convex lens with a 50 mm focal length and 25.4 mm diameter, while the unscattered light was blocked by a cylindrical metal rod with a 3 mm diameter placed at the focal plane of the lens. The collected light was converted into current by a silicon photodiode (S2386-44K, Hamamatsu, Japan). Then, the photocurrent was converted to voltage via a transimpedance amplifier where the photodiode was operating in photovoltaic mode with no external bias and the feedback resistor was 200 kΩ. Finally, the voltage was amplified by 8 times via an op-amp circuit, and then digitalized by an A/D converter. The digital signal was uploaded to a PC through a serial port and analyzed by Microsoft Excel program. The photograph of the nephelometric system is shown in Figure S1.

### 2.2. Immunoagglutination Assay for CRP Detection

Polystyrene beads with a diameter of about 100 nm were used as latex particles to enhance the sensitivity of immunoagglutination assay. The surface of particles was functionalized with carboxyl groups so that proteins could be immobilized on the beads. Anti-CRP polyclonal antibodies were incubated with carboxylated latex particles to form covalent bond and these particle-antibody complexes were used as latex reagents. The CRP samples were prepared with varying concentrations ranging from 0 mg/L to 50 mg/L to validate the performance of our nephelometric system. The blank sample contained 1% of bovine serum albumin. The concentration of CRP samples was confirmed by a commercial CRP assay kit (Sekisui Medical, Tokyo, Japan) using an automated biochemistry analyzer (Model 3100, Hitachi, Tokyo, Japan).

**Figure 2:**
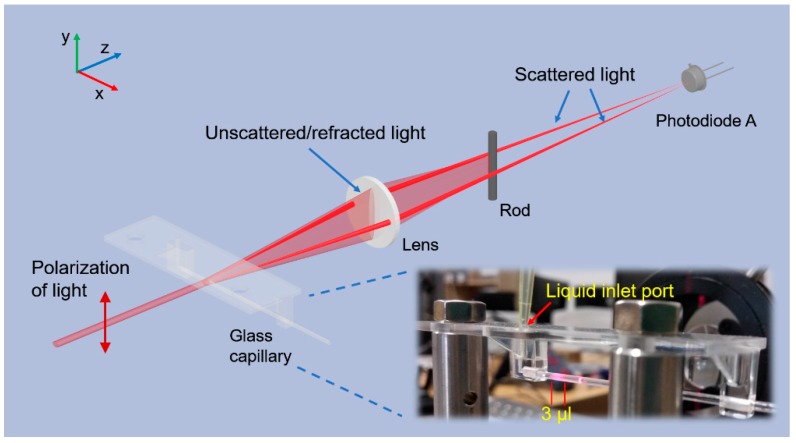
The 3D image of key components of nephelometric system. Only parts of scattered light are drawn. Inserted photograph shows the real image of glass capillary and its mount.

For immunoagglutination assay, the CRP samples were first diluted 230 times with dilution buffer containing ~2% of polyethylene glycol to enhance the rate of immunoaggregation [12]. Then, the diluted sample was mixed with latex reagent at a ratio of 10:1, and the final mixture of a volume of 20 µL was injected into a glass capillary (Micro Haematocrit Tubes, Vitrex, Herlev, Denmark), with inner diameter of 1.15 mm and outer diameter of 1.55 mm, for nephelometry. The glass capillaries were single-use and fixed within the optical system by a homemade mount. The mixtures of the samples and the reagents were injected into the capillary via a liquid inlet port using a pipette, as shown in Figure 2.

## 3. Results and Discussion

### 3.1. System Design

In our nephelometric system, we chose a 670 nm wavelength laser module as light source for two reasons. First, red laser diodes are portable and cost-effective because they have been massively produced for consumer and industrial electronics, such as laser pointers and bar code readers. Second, since the absorption coefficient of oxyhemoglobin in red blood cells has a minimum around 670 nm, we are able to directly monitor the immunoagglutination process in lysed whole blood via 670 nm wavelength laser. This is of significant advantage for point-of-care testing when separation of serum/plasma from a whole blood sample is unfavorable.

In nephelometric immunoagglutination assays, especially latex-enhanced assays, forward detection methods are usually employed because large particles scatter more light in forward direction than backward direction according to Mie scattering theory [11]. Furthermore, it was reported that detection of scattered light at small angle (9.8°) in forward direction could have higher light-scattering amplification, i.e. higher sensitivity, than detection at large angle (40.2°) [21]. Therefore, a small-angle forward detection scheme is preferred for nephelometric immunoassays. However, the unscattered light may interfere with the signal of scattered light when the detection angle is too small. In this work, we proposed a novel design which could not only effectively block the unscattered light but also collect the light scattered at angle as low as 2°. In our system, the glass capillary itself refracted the light in y-z plane due to its cylindrical shape as shown in Figure 2, while the turbulent mixture of sample and reagents in capillary scattered the light in all directions. Both refracted and scattered light were collected by a plano-convex lens with a 50 mm focal length. Since the refracted light only propagated in y-z plane, an opaque cylindrical rod was placed parallel to the *y* axis to effectively block refracted light, which is illustrated in Figure 2. Furthermore, the rod was placed at the focal plane of the lens as a spatial filter so that we could accurately select the angle of light to be blocked. Specifically, using the paraxial approximation, all the light propagating at the same angle will converge to a single point at focal plane of a lens, and an opaque circular disk with a diameter of *d* at the focal plane will block light with incident angle less than arctan (*d*/2*f*), where *f* is the focal length of lens. In our system, we placed a rod with a diameter of 3 mm at the focal plane, meaning that the light with incident angle less than 1.7° would be blocked. This configuration could effectively filter out the unscattered laser light with divergence angle less than 1°. To implement a small-angle detection scheme, the glass capillary was placed 75 mm away from the lens, as shown in Figure 1, so that the lens with a diameter of 25.4 mm could only collect the scattered light with incident angle less than 9.6°. Overall, we developed a nephelometric system which could detect the light scattered at angle ranging from 1.7° to 9.6°, and this system could have a good diagnostic sensitivity due to this small-angle detection scheme. In addition, a silicon photodiode was placed conjugated to glass capillary so that the scattered light could converge to the photodetector, as shown in Figure 2, which could further improve the detection efficiency.

**Figure 3:**
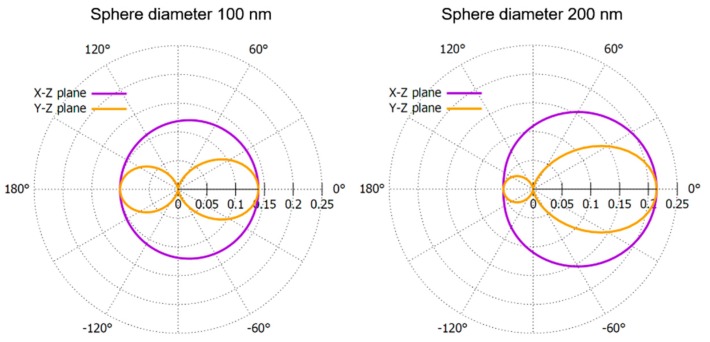
Polar graph of scattering patterns of spheres with diameters of 100 nm and 200 nm. Light is incident from the left on a sphere located at the center of the polar plot. The data are normalized so that the integral of it over 4π steradians is unity.

Light polarization should also be considered in the system design, because the laser diode emitted a polarized laser beam, and scattering properties of particles are dependent on light polarization. To quantitatively illustrate the relation between scattering properties and light polarization, the relative scattering intensity of spheres with diameters of 100 nm and 200 nm were calculated using Mie scattering calculator (website: https://omlc.org/calc/mie_calc.html). The results are shown in Figure 3, assuming that the polarization of light was parallel to *y* axis, as shown in Figure 2, and the refractive indexes of sphere and medium (water) are 1.5 and 1.33, respectively. Obviously, more light is scattered in x-z plane, where the light is detected, than in y-z plane where the light is blocked by the opaque rod. Therefore, the polarization of light in our system was configured to be parallel to *y* axis so that more scattered light could be detected by the photodiode. In addition, as the latex beads aggregate from 100 nm to 200 nm, the scattering pattern becomes more forward directed. This again indicates that the small-angle forward detection scheme could have better performance in nephelometric system.

Low-cost glass capillaries are commonly used for blood collection in clinical applications. In our system, we used them as single-use detection cuvettes, which could effectively prevent cross contamination between test samples. Since a laser beam with a diameter of 3 mm was incident to the capillary with an inner diameter of 1.15 mm, the detection volume of solution in capillary was as low as 3 µL. Such a low detection volume can significantly reduce the required volume and cost of reagents, which is critically valuable when the source materials for immunoassays, such as antibodies, are rare or expensive. In addition, in our design, we only used one laser ($3 US dollars), one plano-convex lens ($8), one opaque rod ($1) and one photodiode ($8) as key components to realize this nephelometric system without any costly parts such as spectrometers and avalanche photodiodes. The total cost of these components is as low as 20 US dollars, which is favorable for widespread application, especially for regions with limited resources.

### 3.2. Quantitative CRP Detection

CRP is a protein made by liver and sent into bloodstream in response to inflammation. Doctors might order a CRP test to check for inflammation of patients, which can indicate infection, tissue injury, and inflammatory disorders [22]. To validate the performance of our nephelometric system, a series of immunoagglutination experiments were conducted to quantitatively measure the concentration of CRP. The mixture of CRP sample, dilution buffer and latex reagent was injected into a glass capillary and its light scattering signal was monitored by our system. After the mixing of CRP samples and latex reagents, the CRP and antibody-coated particles will aggregate with increasing diameter. This change of the particle size results in a dramatically increase in the scattered light [11]. Furthermore, as the diameter of particles increases, the scattering pattern becomes more forward directed as shown in Figure 3. Therefore, the forward detected light scattering signal will increase during the immunoagglutination process. The more CRP is in the solution, the faster and larger signal change we will detect.

**Figure 4:**
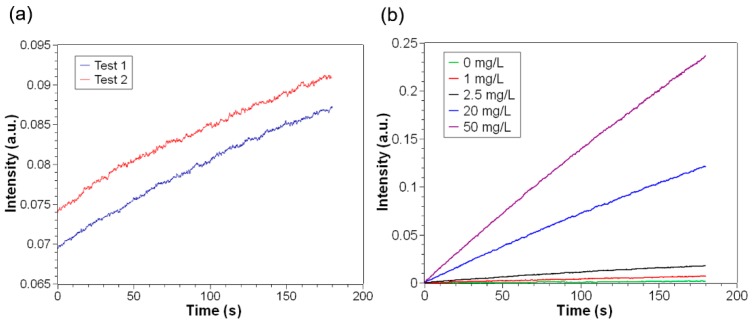
Intensity change of scattered light over time. (**a**) Intensity change of light in two experiments using a same CRP sample (2.5 mg/L) but different capillaries. (**b**) Intensity change of light in experiments using CRP samples with different concentrations. The curves were biased to have the same start point.

As mentioned before, the total detection volume in the capillary was as low as 3 µL. However, it was shown that the collection of the scattered light from low-volume cuvette at small angles could be difficult, since one might collect stray light which was scattered at the glass–air interfaces of the cuvette [23]. We did observe the scattered light at the glass–air interfaces as shown in Figure 4a. The two experiments using a same CRP sample but different capillaries have different signal intensity at the start time point. This could result from the scattering property of glass–air interfaces of two capillaries being different. To reduce the impact of this stray light resulting in a constant bias in signal, one can use kinetic method to determine the rate of signal change in a specified time period, instead of end point method. Obviously, the rate of these two experiments are nearly the same, indicating that the kinetic method can effectively eliminate the impact of stray light.

**Figure 5:**
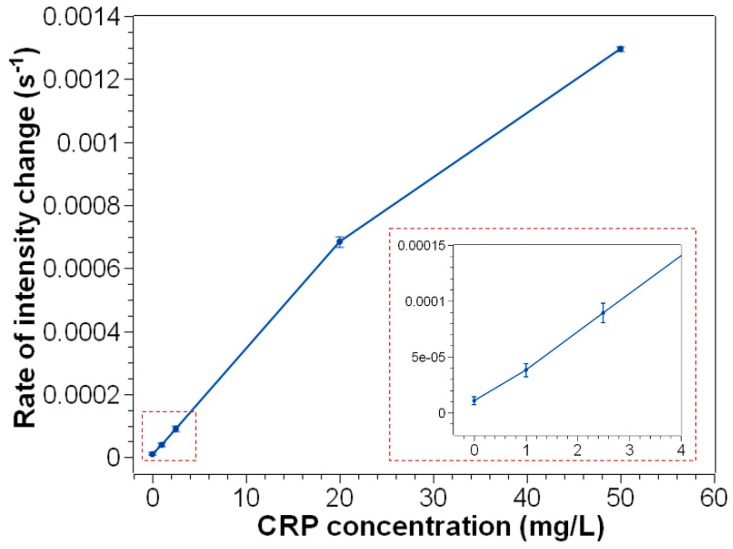
Plot of rate of intensity change versus CRP concentration. The inserted image is a zoomed-in image on low levels of CRP concentration. Error bars represent SD from n = 3 measurements.

Figure 4b displays the intensity of scattered light from solutions with different CRP concentrations plotted as a function of time within 3 min. As we can see, the solutions with different CRP concentrations can be discriminated clearly based on the rate of signal change. Therefore, we can linearly fit the intensity versus time to calculate the slope of line, and establish the relationship between the slope and CRP concentration. The results, also known as calibration curves, are shown in Figure 5. At low antigen concentrations (1 mg/L–20 mg/L), the rate of intensity change increased almost linearly with increasing antigen concentrations. When antigen concentration reached over 20 mg/L, the reactivity became lower. This can be explained by Heidelberger-Kendall curve [11,16], indicating that too much antigen could block the antibody active site, making bridging of antigen and antibody unfavored.

Precision is one of the important performance characteristics for diagnostic tests. The FDA recommend that, for quantitative CRP tests, the assays should have a coefficient of variation (C.V.) less than 10% at clinical cutoff level [22]. We evaluated the precision of the system at CRP concentration of 10 mg/L (cut off level for conventional CRP [22]), and the C.V. of five measurements shown in Table S1 is 3.5%, indicating that our system had a good performance of precision and possessed potential for clinical applications. Another important characteristic for biomolecular sensing is the limits of detection (LOD) which can be simply calculated using following equation [24]

(1) $$\mathrm{LOD}=3*{\mathrm{SD}}_{\mathrm{blank}}/\mathrm{slope}\;\mathrm{of}\;\mathrm{carlibration}\;\mathrm{curve}.$$

According to the equation above, the LOD of CRP sample concentration in our system is 0.37 mg/L. It is comparable to other studies, such as a LOD of 0.18 mg/L using a microfluidic aggregation analyzer [16].

Since the total detection volume of our system is 3 µL and the samples are diluted 253 times, the required CRP sample volume could be as low as 12 nL. Thus, our system needs significantly less total volume than other systems based on turbidimetric or nephelometric immunoagglutination assays, usually with 100–200 µL total volume, without any compromise of system performance. The sample volume requirement of our system is even comparable to other electrical detection systems, in which 2 µL total volume and 50 nL sample volume are needed [16]. This could not only reduce the cost of reagents but also be significantly important when analyte samples are precious. In addition, the total time needed for one CRP test was about four minutes, including one minute for mixing of samples and reagents and three minutes for signal detection. The whole process was homogeneous without any separation and washing steps, and required only liquid mixing which is easy to automate. These are great advantages over ELISA, which usually needs a long reaction time and several washing steps.

## 4. Conclusions

Herein, we proposed a novel low-cost micro-volume nephelometric system for quantitative immunoagglutination assays. The total cost of key components in this system was only about 20 US dollars, and the total detection volume was as low as 3 µL, which further reduced the cost of reagents and required sample volume. We verified the system performance via a quantitative nephelometric CRP assay, and demonstrated that it had similar performance to other systems using optical detection methods, and has high potential to be utilized for clinical diagnostic applications.

In our CRP assay, there is only 12 nL CRP sample in the final detection volume. Precise manipulation of such low volume liquid could be challenging. To fully utilize the advantages of low volume detection, other techniques, such as microfluidics, should be employed to accurately meter and control the liquid. By integrating these techniques, our proposed system will significantly benefit diagnostic tests, especially at point-of-care setting, in which convenience, rapidness and requirement of low sample volume are critical.

## Supplementary Materials

The following are available online at https://www.mdpi.com/1424-8220/19/20/4359/s1, Figure S1: Photograph of the nephelometric system. Table S1: Results of experiments to evaluate the precision of system.
