# Supplementary material for: A Low-Cost Micro-Volume Nephelometric System for Quantitative Immunoagglutination Assays
Source: Sensors (Basel). 2019 Oct 9;19(20):4359. doi: 10.3390/s19204359 (PMC6832725; doi:10.3390/s19204359)
Supplement: Supplementary file 1 [file sensors-19-04359-s001.pdf]

sensors

MDPI

Supplementary Materials

A Low-Cost Micro-Volume Nephelometric System for Quantitative Immunoagglutination Assays

Qiqi Sun [1,2,*], Wei Zheng [1], Chao Lin [2] and Dongxuan Shen [2]

[1] Research Laboratory for Biomedical Optics and Molecular Imaging, Shenzhen Key Laboratory for Molecular Imaging, Shenzhen Institutes of Advanced Technology, Chinese Academy of Sciences, Shenzhen 518055, China; zhengwei@siat.ac.cn

[2] Edan Instruments, Inc., Shenzhen 518067, China; linchao@edan.com.cn (C.L.); dshen@edan.com.cn (D.S.)

* Correspondence: qsunaa@connect.ust.hk

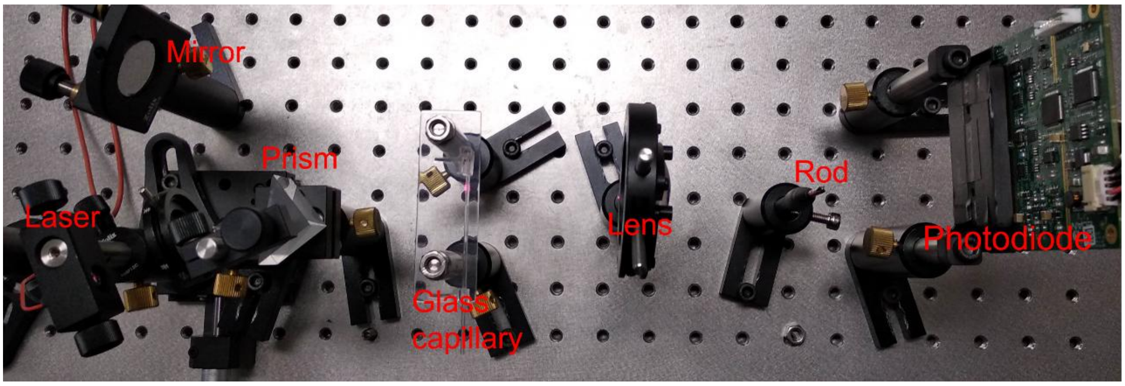

Figure S1. Photograph of the nephelometric system.

Table S1. Results of experiments to evaluate the precision of system.

Experiment No.	Signal Change Rate (s^{-1})
1	0.000307782
2	0.000318123
3	0.00029997
4	0.000316522
5	0.000329339
Average	0.000314347
STD	1.11079E-05
C.V.	0.035336254
